# Effect of Age and Cytoskeletal Elements on the Indentation-Dependent Mechanical Properties of Chondrocytes

**DOI:** 10.1371/journal.pone.0061651

**Published:** 2013-04-16

**Authors:** Nadeen O. Chahine, Craig Blanchette, Cynthia B. Thomas, Jeffrey Lu, Dominik Haudenschild, Gabriela G. Loots

**Affiliations:** 1 The Feinstein Institute for Medical Research, Hofstra North Shore LIJ School of Medicine, Manhasset, New York, United States of America; 2 Lawrence Livermore National Laboratory, Physical and Life Sciences Directorate, Biosciences and Biotechnology Division, Livermore, California, United States of America; 3 Lawrence J. Ellison Musculoskeletal Research Center, Department of Orthopaedic Surgery, University of California Davis Medical Center, Sacramento, California, United States of America; 4 School of Natural Sciences, University of California Merced, Merced, California, United States of America; Dalhousie University, Canada

## Abstract

Articular cartilage chondrocytes are responsible for the synthesis, maintenance, and turnover of the extracellular matrix, metabolic processes that contribute to the mechanical properties of these cells. Here, we systematically evaluated the effect of age and cytoskeletal disruptors on the mechanical properties of chondrocytes as a function of deformation. We quantified the indentation-dependent mechanical properties of chondrocytes isolated from neonatal (1-day), adult (5-year) and geriatric (12-year) bovine knees using atomic force microscopy (AFM). We also measured the contribution of the actin and intermediate filaments to the indentation-dependent mechanical properties of chondrocytes. By integrating AFM with confocal fluorescent microscopy, we monitored cytoskeletal and biomechanical deformation in transgenic cells (GFP-vimentin and mCherry-actin) under compression. We found that the elastic modulus of chondrocytes in all age groups decreased with increased indentation (15–2000 nm). The elastic modulus of adult chondrocytes was significantly greater than neonatal cells at indentations greater than 500 nm. Viscoelastic moduli (instantaneous and equilibrium) were comparable in all age groups examined; however, the intrinsic viscosity was lower in geriatric chondrocytes than neonatal. Disrupting the actin or the intermediate filament structures altered the mechanical properties of chondrocytes by decreasing the elastic modulus and viscoelastic properties, resulting in a dramatic loss of indentation-dependent response with treatment. Actin and vimentin cytoskeletal structures were monitored using confocal fluorescent microscopy in transgenic cells treated with disruptors, and both treatments had a profound disruptive effect on the actin filaments. Here we show that disrupting the structure of intermediate filaments indirectly altered the configuration of the actin cytoskeleton. These findings underscore the importance of the cytoskeletal elements in the overall mechanical response of chondrocytes, indicating that intermediate filament integrity is key to the non-linear elastic properties of chondrocytes. This study improves our understanding of the mechanical properties of articular cartilage at the single cell level.

## Introduction

Articular cartilage is the connective tissue that lines the ends of bones in diarthrodial joints and provides a low-friction bearing surface for the transmission and distribution of mechanical loads in the skeleton. Chondrocytes are the dominant cell type found in articular cartilage, and these cells are responsible for the synthesis, maintenance, and gradual turnover of the extracellular matrix (ECM) [1]. The ECM is composed primarily of a hydrated matrix containing mostly type-II collagen and highly charged proteoglycan (PG) molecules. The composition and architecture of the matrix and the tissue’s high water content (70–80% of wet weight) enable cartilage to withstand complex compressive, tensile and shear forces in joints [2], [3]. The cartilage ECM has been shown to remodel in response to the functional demands of mechanical loading, and this process is mediated through the metabolic activity of chondrocytes [1].

Articular cartilage is a functionally heterogeneous tissue such that the position of a chondrocyte relative to the joint surface and the bone interface defines its extracellular and intracellular biochemical composition [4], [5] resulting in zonal depth-dependent mechanical properties [6]–[10]. The application of mechanical loads during locomotion results in non-uniform deformation patterns throughout the tissue, which can expose chondrocytes to a range of deformation magnitudes that is in part dependent on the zonal location [11]–[13]. For example, the superficial zone has been shown to possess lower compressive stiffness than the deep zone, resulting in larger deformation magnitudes in the superficial zone relative to the middle or deep zone [14]. Chondrocyte biosynthesis is tightly regulated by the profile, frequency, magnitude and duration of the applied load or deformation [15]–[18]. Compression of cartilage to physiological strain magnitudes serves as a signal for modulating chondrocyte biosynthetic activity through the depth of cartilage, while prolonged compression at excessive strains may be responsible for tissue and cellular damage [15]–[18]. Indeed, certain zonal changes in metabolic activity have been correlated with zonal strain magnitudes, suggesting that zone-specific variations in mechanical stimuli could be responsible for spatially varied patterns of cartilage metabolic activity under load [19].

The mechanical response of a cell to loading is dependent on the morphology, mechanical properties, and cell-matrix interactions; properties that are heavily influenced by the cytoskeleton. The cytoskeleton is composed of three interconnected filament systems: actin, microtubules and intermediate filaments (IFs) that contribute to a cell’s shape, structural integrity and movement. Among the components of the cytoskeleton, both actin and IFs [20]–[22], have been postulated to contribute to the mechanical properties of cells. The contribution of the actin cytoskeleton to the mechanical properties of chondrocytes has been widely studied [23]–[25]. However, less is known about the role of IFs in this process. IFs comprise a large family of proteins that share a common tripartite domain structure and have the ability to assemble into 8–12 nm wide filaments [26]. By examining the cytoskeletal architecture of chondrocytes in 3D cultures, it was recently reported that vimentin filaments represent a less dynamic and more rigid structure relative to the actin filaments, which suggests that IFs may play an important role in the overall mechanical properties and response of chondrocytes [22]. The use of chemical entities that specifically disrupt actin and IFs was previously shown to result in a significant decrease in the mechanical stiffness of human chondrocytes [27]. However, in these reports the mechanical stiffness both in the absence and presence of cytoskeletal disruptors was only measured under a single loading condition. Consequently, the mechanical properties of the chondrocyte as a function of deformation (i.e. indentation-dependent mechanical properties) were not systematically evaluated. In addition, the effect of chemical disruption treatment on the microstructure of the cytoskeleton was not examined.

Articular cartilage deteriorates with age from wear and tear, trauma and as a function of disease, which can ultimately result in the development of osteoarthritis. Articular damage has been shown to be associated with a loss of anabolic activity in aged tissue [28], i.e. the ECM is no longer efficiently maintained, which implies that chondrocytes may exhibit altered mechano-responsive properties as they age. In addition, chondrocytes in mature cartilage are less vulnerable to load-induced injury than those in immature cartilage [29], despite decreases in the biomechanical properties of ECM with age [30], [31]. While previous studies have examined the effect of age on human and rabbit chondrocyte mechanical properties [27], [32], [33], findings from these studies demonstrated a variety of trends that are dependent on species and/or methods of testing. Thus, there is a clear discrepancy in the reported effects of aging on the biomechanical properties of chondrocytes.

The goal of this study was to measure the indentation-dependent mechanical properties of chondrocytes isolated from neonatal (1-day), adult (5-year) and geriatric (12-year) bovine articular cartilage using atomic force microscopy (AFM). Chondrocytes derived from bovine joints represent one of the most widely used model systems for cartilage tissue engineering and chondrocyte mechanobiology. In addition, we also systematically measured the contributions of the actin and IFs on the indentation-dependent mechanical properties of chondrocytes. Furthermore, to better understand the contribution of the cytoskeleton to indentation-dependent behavior, we utilized an integrated AFM and confocal fluorescent microscopy system to simultaneously acquire high resolution fluorescent images of transfected (GFP-vimentin and mCherry-actin) chondrocytes under compression. Here, we show that the elastic modulus of chondrocytes decreases nonlinearly with increased indentation. In addition, a significant increase in stiffness was observed in adult chondrocytes relative to neonatal cells, at indentations greater than 500 nm. The viscoelastic analyses demonstrated that the intrinsic viscosity was statistically greater in neonatal compared to geriatric chondrocytes. We also systematically evaluated the effects of the actin and IFs on the indentation dependent mechanical properties of chondrocytes and found that disrupting either the actin or the IFs alters the mechanical properties of chondrocytes by decreasing the elastic modulus and the viscoelastic properties.

## Results and Discussion

### Effect of Age on the Indentation-dependent Mechanical Stiffness and Viscoelastic Properties of Chondrocytes

**Figure 1:**
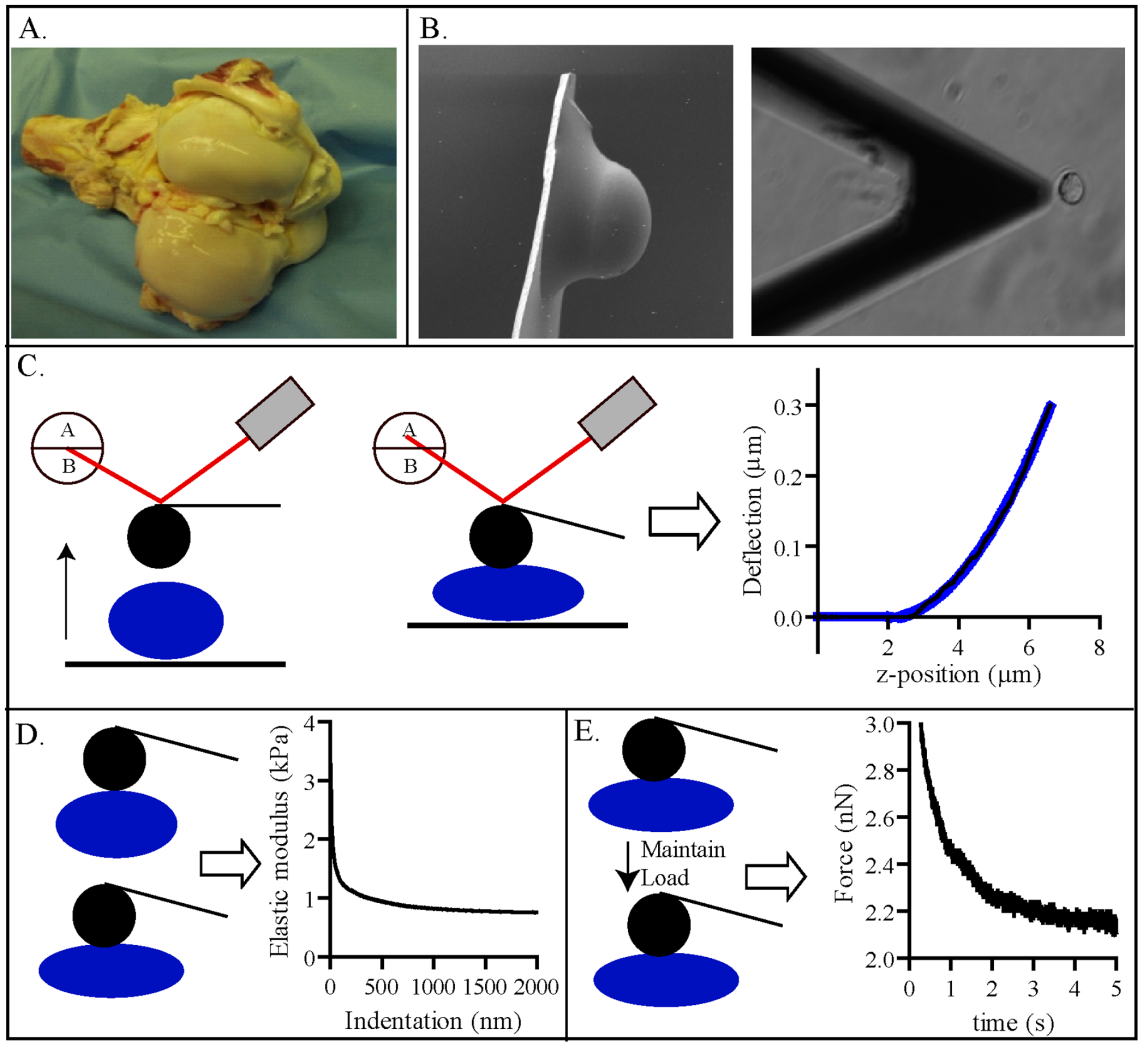
Experimental set up. Image of bovine joint used for chondrocyte isolation **(A)**. Transmission Electron Microscopy (TEM) image of the Atomic Force Microscopy (AFM) tip/bead and image of the alignment of tip/bead over a single chondrocyte **(B)**. Schematic of measuring Force vs chondrocyte indentation using AFM **(C)**. Schematic and representative indentation-dependent elastic modulus of an individual chondrocyte **(D)**. Schematic of the approach used to acquire viscoelastic properties of individual chondrocytes and a representative viscoelastic force curve from an individual chondrocyte **(E)**

**Figure 2:**
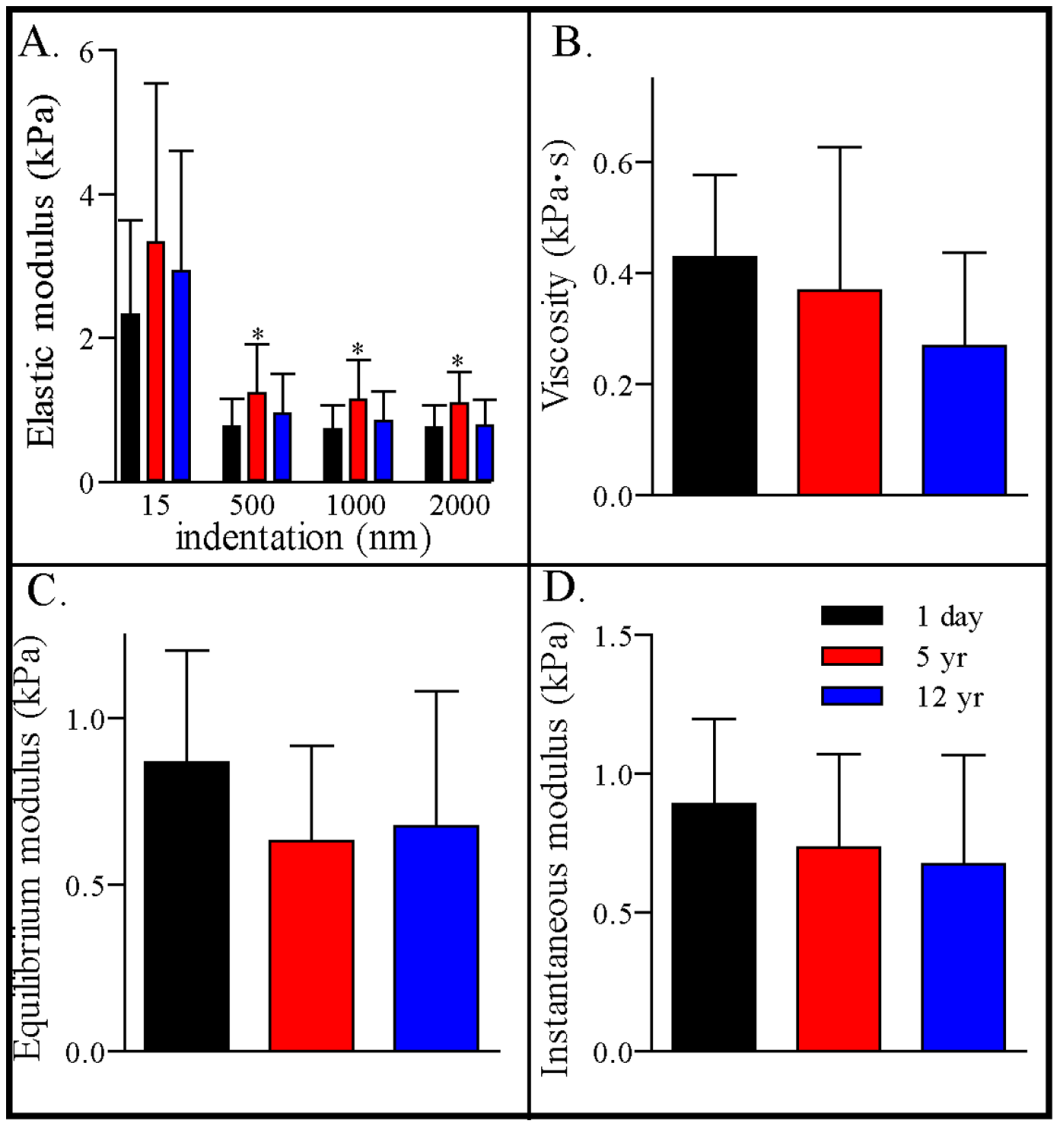
The effect of age on the viscoelastic properties of bovine chondrocytes. Comparing indentation-dependent elastic modulus **(A)**, viscosity **(B)**, equilibrium modulus **(C)** and instantaneous modulus **(D)** of chondrocytes isolated from different aged bovine [1 d (black bars), 5 yr (red bars) and 12 yr (blue bars)]. *p<0.05 vs. 1 d (within indentation).

Chondrocytes isolated from neonatal, adult and geriatric bovine articular cartilage were examined under increasing levels of indentation to determine their point-wise elastic modulus as a function of deformation, in each age group (Figure 1D). Our findings indicate that the apparent modulus decreased nonlinearly with increasing indentation. The modulus was greatest at smallest indentation, and decreased monotonically with increased compression. In neonatal cells, the apparent modulus at the early indentation point of 15 nm (E_15_) was found to be 2.32±1.3 kPa, and decreased to a constant value after a critical indentation (D*) of 260±170 nm. As the indentation increased beyond D*, E_i_ was found to be constant, exhibiting a value of 0.75±0.3 kPa between an indentation of 500 and 2000 nm (Figure 2A). These findings indicate that the chondrocyte elastic modulus is dependent on the magnitude of indentation, which may reflect alterations in the structural state of the cell. The elastic moduli measured in this study at a 1500 nm indentation were comparable to previously reported studies, which measured the modulus of chondrocytes from porcine cartilage at a ∼15% strain [34]. There have been a few previous studies that also examined the nonlinear elastic response of cells [35], [36]. Costa *et al*. analyzed the point-wise indentation-dependent elastic modulus of human aortic and bovine lung endothelial cells and found that the elastic modulus increased (∼0.5 kPa to ∼7 kPa) as the indentation depth was increased 100–600 nm [36]. This trend and range was quite different than that what was observed in the current study, possibly due to the fact that flattened cells were tested using a nano-indentor as opposed to our study that used a micro-indentor to test cells with a rounded morphology [36]. Interestingly, when Azeloglu and Costa examined the indentation-dependent elastic modulus of neonatal rat cardiac myocytes under systolic and diastolic conditions, the modulus only displayed an increase under systolic conditions and a slight decrease under diastolic conditions, which was attributed to the contractile properties of the cell under the systolic conditions [35].

Qualitatively similar non-linear responses in E_i_ were observed in chondrocytes from all age groups (Figure 2A), with a higher modulus measured at small indentation that decreased to a relatively constant value at indentations greater than 500 nm. The elastic modulus measured at small indentation (E_15_) was comparable across all age groups (p>0.8). The elastic modulus measured at 500, 1000 or 2000 nm indentation exhibited some age-dependent changes. E_500_, E_1000,_ E_2000_ in adult (5-yr old) cartilage was found to be significantly greater than that measured in neonatal chondrocytes (Figure 2A, p<0.05). Critical indentation, D* in chondrocytes from 12-yr old cartilage (584±206 nm) was found to be significantly greater than that measured in 1-day old chondrocytes (p<0.01). No significant difference in D* was observed in 5-yr old chondrocytes (404±274 nm) in comparison to other age groups. While the elastic modulus of the adult chondrocytes was found to be significantly greater than neonatal chondrocytes at indentations greater than D*, no significant differences in the elastic modulus between age groups were observed at very small indentations.

The effect of age on the mechanical stiffness of chondrocytes has been examined previously in other model systems. Steklov *et al*. reported that the stiffness of chondrocytes obtained from older human individuals (>55 years old) was higher than chondrocytes obtained from younger human individuals (18–35 years old) [27]. Our results are consistent with the overall findings of that study; chondrocytes exhibit increased stiffness with age. In bovine, this increase occurs between neonatal (1-day) and adulthood (5-yrs), however further aging into geriatric years was not associated with increased mechanical stiffness relative to younger cells in our study. Hsieh *et al*, found that human OA chondrocytes had an overall lower mechanical stiffness than normal chondrocytes [32]. Thus, it is possible that the reduction in elastic properties of geriatric bovine chondrocytes may be associated with further aging and degenerative changes, nevertheless our cells were isolated from macroscopically normal looking tissue; however the thickness of the cartilage was diminished relative to younger cartilage. It is also worth noting that the effect of aging on mechanical stiffness has been examined in other cell types. Notably, Zahn *et al*. reported that the Young’s modulus of fibroblasts obtained from young human donors (<25 years) was higher than that of fibroblasts obtained from older human donors (>30 years) [37]. The variability in the literature regarding the effect of age on chondrocyte mechanical stiffness may also be related to the different cellular function and model systems.

The effect of age on the viscoelastic properties of bovine chondrocytes was also evaluated. In these experiments, the cells were probed to an indentation depth of 1500 nm (∼15% deformation relative to cell diameter) and viscoelastic relaxation was measured over a 10 second (s) period of time (Figure 2B). The instantaneous modulus was found to be comparable across all age groups (Figure 2B). The equilibrium moduli was also comparable in all age groups [Neonatal: 0.87±0.3 kPa; Adult: 0.63±0.3 kPa (p = 0.07); Geriatric: 0.63±0.4 (p = 0.06)], such that neonatal chondrocytes had a higher equilibrium modulus relative to the two older groups (Figure 2C). The intrinsic viscosity (μ) was found to be statistically greater in neonatal (0.43±0.1 kPa.s) compared to aged chondrocytes (0.27±0.1 kPa.s, p<0.05, Figure 2D). A recent study on chondrocytes from healthy rabbit cartilage reported that significantly lower E_O_, E_Y_ and μ were observed in cells isolated from old (31 month) relative to young (2 month) or adult (8 month) animals [33]. Our findings are consistent with this previous study, although the age between cows and rabbits cannot be directly correlated. The aforementioned study and the current findings are however different than the study by Steklov *et al*. where lower E_O_, E_Y_ and μ values were observed in normal human chondrocytes from adult (18–35 year) relative to normal chondrocytes from aged (55+) adults [27].

There are several plausible explanations for these discrepancies. First, the chondrocytes used in this study were obtained from bovine tissue, and it is likely that the effect of healthy aging on the mechanical properties of chondrocytes is species-specific and dependent on the age of the animal relative to the average lifespan. Second, a different type of testing was previously used (micropipette aspiration) to ascertain viscoelastic properties. While other studies have shown AFM and micropipette aspiration to yield comparable results [34], the use of a micropipette with an inner diameter of ∼5–6 µm deforms a smaller region of the cell compared to the 10 µm bead we used in the current study. Moreover, the micropipette technique applies a constant pressure on the cell resulting in the equivalent of a single step load of ∼70 kPa, while AFM measures the properties of the cell at a relatively lower indentation force (∼2.5 nN, ∼0.5 kPa). Based on our findings that chondrocytes exhibit indentation-dependent properties (Figure 2A), it is plausible that the properties represent behaviors in different regions on the deformation curve, yielding different responses with aging. Finally, it is likely that we did not observe any significant differences due to the heterogeneity inherent in the cellular properties of chondrocytes isolated from different zones of the full thickness bovine cartilage harvested from knee joints [34].

The differences we observed in the mechanical properties of the chondrocytes isolated from different aged cartilage, and the indentation-dependent response we observed at all age groups have implications regarding the mechanobiology of chondrocytes. The decrease in viscosity of the cell as a function of age is indicative of a shift in the cellular behavior away from viscoelasticity toward a more elastic behavior. This shift is associated with loss in energy dissipation of the cell, and may potentially increase the apparent stress concomitant with cellular deformation. The lower modulus and increased permeability of osteoarthrotic ECM result in greater and more-rapid deformation of the tissue than normal [30], [38]. The changes in the mechanical (elastic and viscoelastic) properties of the cell with respect to age described herein, in combination with ECM changes associated with aging, may influence the synthetic activity of the chondrocytes, which are known to respond to their mechanical environment [17], [19], [39]. Previous studies have also demonstrated that degradation of articular cartilage is associated with incongruity, instability and elevation in joint contact stress [40]. Computational models of cellular stress and strain indicate that cellular responses are dependent on the applied load, cell morphology, and relative mechanical properties of the extracellular and pericellular matrix to the cell properties [41]–[43]. Increased joint loads associated with aging may also increase the level of deformation propagated to the cellular microenvironment. In combination with higher elastic modulus and lower apparent viscosity with aging (Figure 2B–D), these factors may result in altered cellular deformation relative to chondrocytes in young joints. In addition, the potential increase in tissue deformation associated with aging may shift chondrocyte mechanical behavior to a softer region in the indentation-dependent response; thus, altering the mechano-sensitivity of the cell. While our findings are consistent with this hypothesis, further experiments are required to examine the relationship between the mechanical properties of the cell, mechanosensitivity, and cellular stress and strain in the microenvironment of the cell.

### Effect of Cytoskeletal Elements (Actin and IFs) on the Elastic and Viscoelastic Properties of Chondrocytes

To assess the effects of actin and IFs on the elastic modulus and viscoelastic modulus, the same experiments described above were performed on chondrocytes obtained from 1 day old bovine using chemical compounds that specifically disrupt actin (cytochalasin B) [44] and IFs (acrylamide) [25]. Cytochalasin compounds disrupt actin filaments through a multi-step mechanism, where actin filaments are capped by binding to the barbed end of a growing filament [45]; then cleaved [46]; and stimulated by increased ATPase activity [47]. Acrylamide treatment has been shown to inhibit IFs without causing any significant effects on protein synthesis or cell metabolism [48]. Previously, it has been shown that acrylamide treatment causes IFs, including vimentin, to collapse into juxta-nuclear aggregates and both microtubules and actin-containing stress fibers were found to be unaffected by the acrylamide treatment [16]. Thus, cytochalasin B (cytoB) and acrylamide were used to selectively disrupt actin and IFs, respectively, and the effects of this disruption on the indentation-dependent elastic modulus and the viscoelastic properties were quantified.

**Figure 3:**
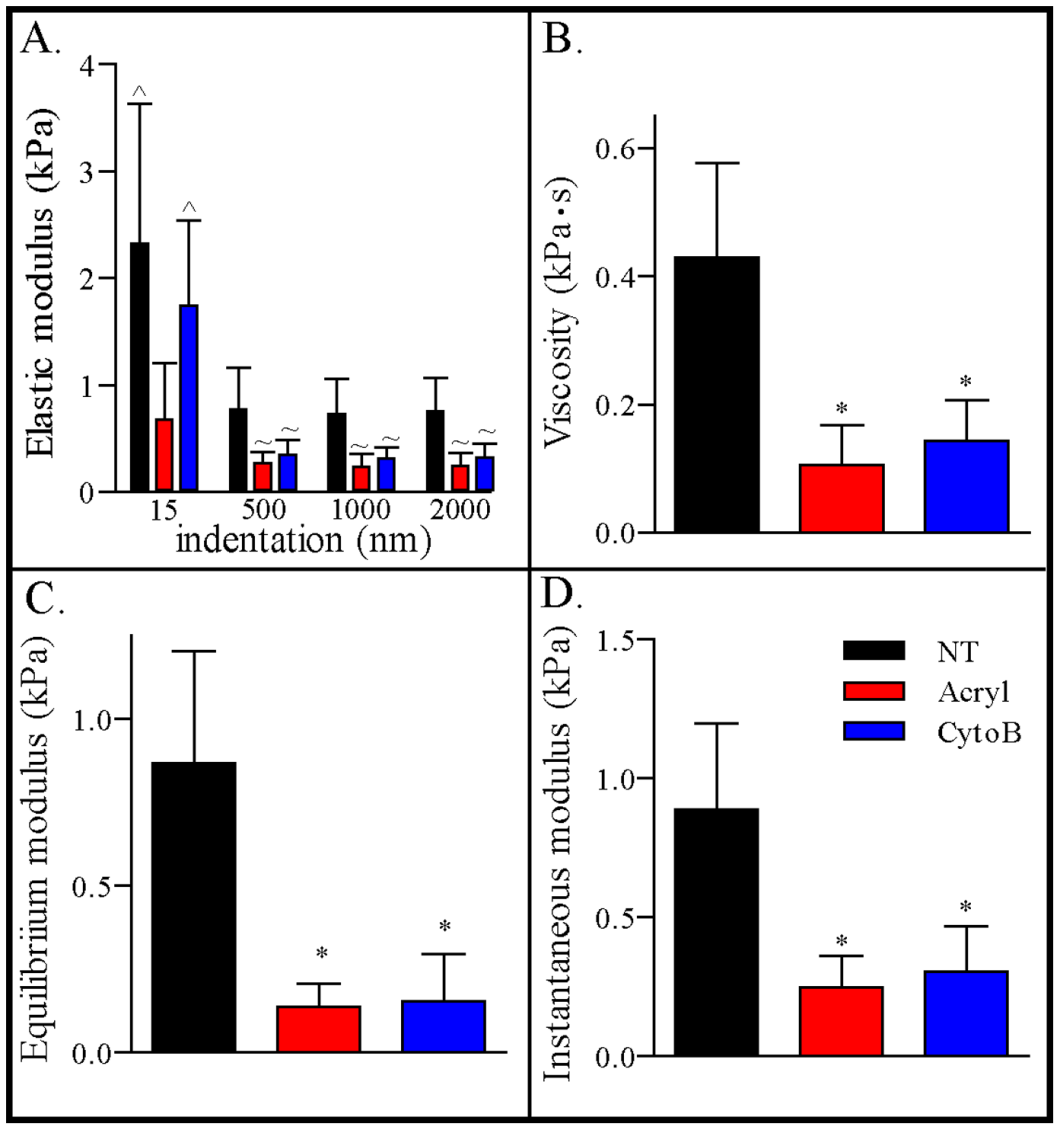
Indentation-dependent viscoelastic properties of chondrocytes are affected by cytoskeletal disrupters. Comparing indentation dependent elastic modulus **(A)**, viscosity **(B)**, equilibrium modulus **(C)** and instantaneous modulus **(D)** of chondrocytes subjected to different treatment conditions [NT (control; no treatment), Acryl (acrylamide), CytoB (cytochalasin B)]. (*p<0.001 vs. control; ∼p<0.05 vs control; ∧p<0.05 vs Acryl).

The decrease in elastic modulus as a function of indentation was significantly affected by treatment with either acrylamide or cytoB. Upon disruption of the cytoskeleton with acrylamide, the elastic modulus decreased relative to the untreated control at all indentation levels (Figure 3A), yielding relatively constant moduli with increased indentation ranging from 15 to 2000 nm. The indentation-dependent elastic modulus measured with cytoB exhibited some non-linearity, and the E_15_ was found to be comparable to the untreated control (Figure 3A). However, with increasing indentation (>500 nm), the elastic modulus of cells treated with cytoB was significantly lower than untreated controls. E_500_, E_1000,_ E_2000_ in cytoB and acrylamide groups were comparable, and significantly lower than untreated cells (p<0.0001). The effect of cytoskeletal treatment on the mechanical properties of chondrocytes appeared to be dependent on the indentation magnitude, with acrylamide resulting in a reduction in the elastic properties at all indentations. In contrast, cytoB treatment reduced the elastic modulus only at indentations greater than D*. The viscoelastic properties of chondrocytes subjected to acrylamide and cytoB treatment were also measured (Figure 3B–D). E_O_, E_Y_ and μ in chondrocytes treated with acrylamide and cytoB were both significantly lower than the untreated controls (Figure 3B–D, p<0.0001). However, the viscoelastic properties measured in the 2 treatment groups were comparable to each another.

Our findings indicate cytoskeleton disruption affected both the elastic and viscoelastic properties of bovine chondrocytes. Interestingly, we observed a loss of the indentation-dependent behavior in the elastic modulus in the acrylamide treated group (Figure 3B). The disruption of IFs eliminated all indentation-dependent behavior (Figure 3B), further implicating its contribution to supporting the mechanical response of the cell to indentation. This finding also supports the notion that IF and cytoskeletal integrity are key contributors to the non-linear indentation dependent properties of bovine chondrocytes. The effect of disruption of both actin and IFs using cytoB compounds and acrylamide, respectively, on the mechanical properties of human chondrocytes has been previously reported using micropipette aspiration [25], where similar trends were observed for actin disruption with cytoB. In the aforementioned study however, treatment of chondrocytes with acrylamide at similar concentration used in this study, did not alter E_O_, E_Y_ or μ. An acrylamide dose greater by one order of magnitude did indeed alter the viscoelastic properties of human chondrocytes [25]. It is worth noting that the effect of cytoB treatment on the mechanical stiffness of cells has also been examined with other cell types. Sokolov *et al.* found that treatment of primary human epithelial cells with cytochalasin B significantly decreased the Young’s modulus of cells that had been passaged 8 to 12 times [49]. Our results demonstrate that disrupting either the actin or the IF cytoskeletal structures alters the mechanical properties of chondrocytes by decreasing the elastic modulus and the viscoelastic properties, and that IF integrity is a key contributor to non-linear elastic modulus of chondrocytes.

### Effect of Cytochalasin B and Acrylamide on Actin and Vimentin Organization in the Presence and Absence of an Applied Load

**Figure 4:**
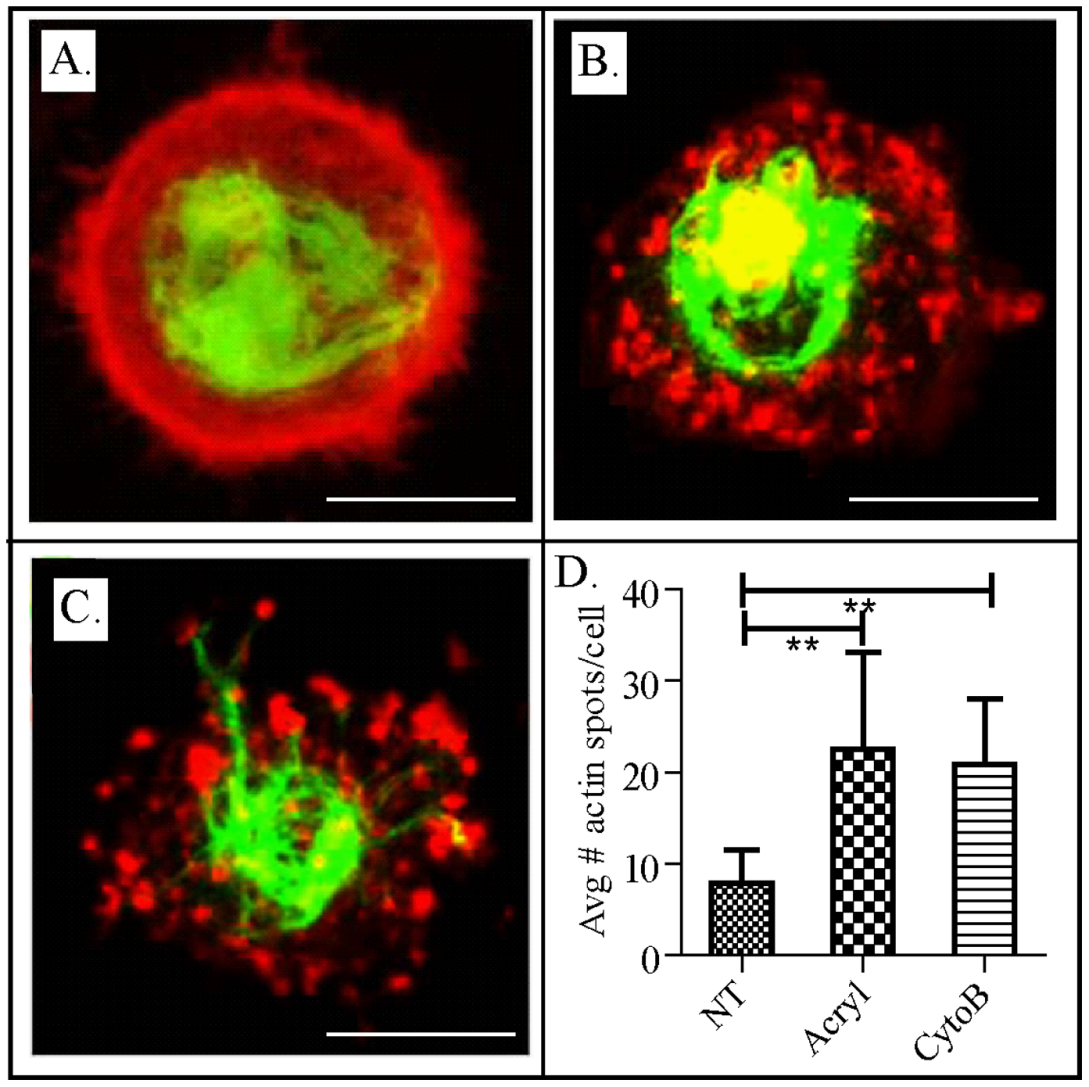
Visualizing the effects of cytoskeletal disrupters on chondrocytes. Confocal fluorescent images of chondrocytes treated with media **(A)**, 5 mM acrylamide for 16 hours **(B)** and 5 µM cytochalasin B for 1 hour **(C)**. Scale bar –5 µm. Analysis of actin aggregates per chondrocyte **(D)**. More than 20 individual cells per treatment were analyzed using Image J. Asterisks indicates a significant difference (p<0.005).

To better understand the effect of cytoB and acrylamide treatment on the overall microstructure of the cytoskeleton, chondrocytes were transfected with mCherry-actin and GFP-vimentin to monitor changes in actin and vimentin microstructures using fluorescence. In the absence of cytoskeletal disruptors, the mCherry-actin appeared relatively diffuse and uniform with a more dense red fluorescent intensity observed near the edge of the cell (Figure 4A). The vimentin-GFP was predominantly present at the center of the cell and appeared very dense and compact with distinctly visible filaments (Figure 4A), consistent with previous reports [50], [51]. When chondrocytes were treated with cytoB, the mCherry-actin ring structure disorganized into a highly punctate configuration, with bright fluorescent spots present throughout the cell (Figure 4B). No dramatic effects on the GFP-vimentin structures were observed. This morphology is highly consistent with the expected dissolution of the actin filaments. Surprisingly, a similar morphological change was observed when the chondrocytes were treated with 5 mM acrylamide (Figure 4C). Under these conditions, the mCherry-actin appeared as punctate spots throughout the cell and the GFP-vimentin structure remained relatively unchanged. This was unexpected since previous studies of similar acrylamide treatments have been reported to only affect IFs structure [48], [52].

There are two possible explanations for the observed results: 1) acrylamide has a direct effect on actin filaments, or 2) mild disruption of IFs that are below the resolution of our optical system have indirect effects on the actin filaments. Previous studies have demonstrated that acrylamide has no direct effect on actin filaments [48], [52]; thus, we do not believe that the first explanation adequately explains the morphological changes observed after acrylamide treatment. Therefore, it is possible that mild disruption of IFs, of which vimentin may be a major contributor, indirectly resulted in the collapse of the actin filaments. IFs have been shown to be linked to actin microfilaments through plectin protein molecules [53], [54]. This multidomain protein interacts with IFs through a specific binding domain that is located near the carboxyl terminus of the protein [55], whereas the amino-terminal domain of the protein contains an actin-binding domain [56], [57]. Plectin is the primary protein that is involved in coordinating the different cytoskeletal elements and is vital to the overall motility and mechanical properties of individual cells [58]. Although the association between plectin and actin and IFs has been widely established, very little is known about the relationship between the structural integrity of the different filaments when the link between them is lost. The results of this study suggest that mild disruption of the IFs results in a disruption of actin structure, which may have been mediated through a loss of interaction between the two filaments via plectin. A previous study demonstrated a significant decrease in plectin-vimentin interactions when vimentin was under-expressed and no longer formed dense IFs in a glioma cell line [54]; however, the absence of a plectin-vimentin intermediate filament interaction did not result in a collapse of actin filaments, as was observed in this study. Therefore, the effect observed in this study on chondrocytes is qualitatively different from that observed in other cell types. In fact, a similar phenomenon was observed in a previous study, where treatment of chondrocytes with acrylamide was shown to result in a loss of actin structure [25]; however, the potential reason for this change in morphology was not addressed. Although the hypothesis that this collapse of the actin structure was mediated by a loss in the interaction between actin and IFs via plectin is intriguing, further studies are required to elucidate its role.

**Figure 5:**
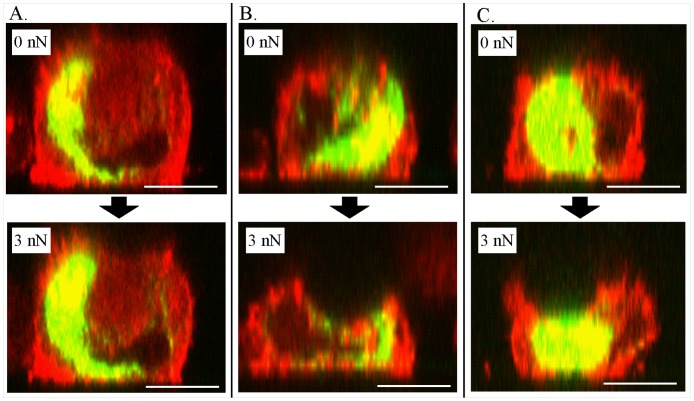
Visualizing the effects of cytosckeletal disrupters on chondrocyte indentation. Z- stacked confocal fluorescent images of chondrocytes compressed at a force of 0 nN and 3 nN treated with media **(A)**, 5 mM acrylamide for 16 hours **(B)** and 5 µM cytochalasin B for 1 hour **(C)**. Scale bar –5 µm.

**Figure 6:**
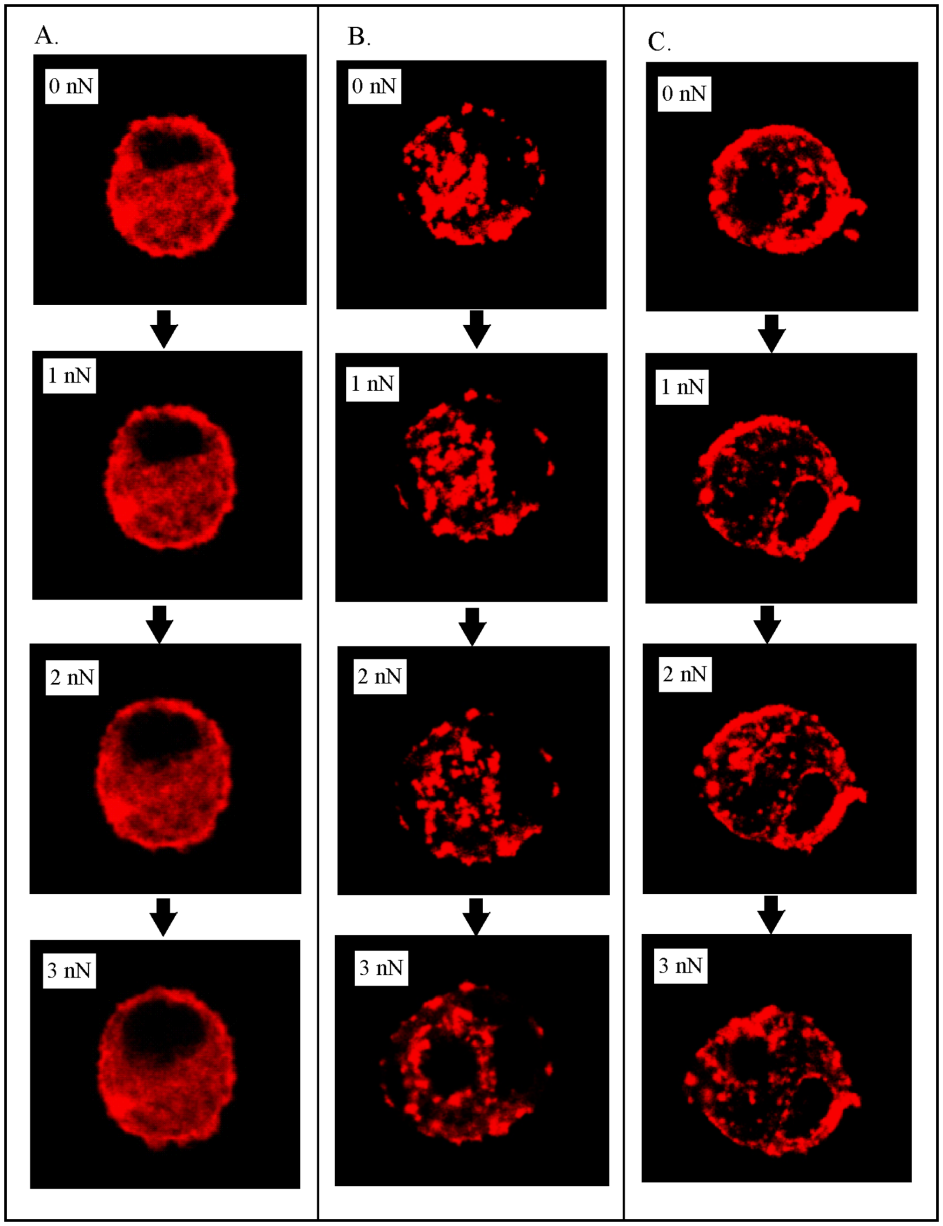
Visualizing the actin cytoskeleton of compressed chondrocytes in the presence of cytosckeletal disrupters. Cross-sectional confocal fluorescent images of chondrocytes compressed at a force of 0, 1, 2 and 3 nN treated with media **(A)**, 5 mM acrylamide for 16 hours **(B)** and 5 µM cytochalasin B for 1 hour **(C)**.

To better understand the effect of treatment on the mechanical response of the cells under load, mCherry-actin and GFP-vimentin transfected chondrocytes were imaged by confocal microscopy during compression. Z stacked confocal microscopy images of single cells under a load of 0 and 3 nN at the different treatment conditions are shown in Figure 5. While a recent study suggested that transfection of cytoskeletal proteins may induce changes in the mechanical properties of the cell, we expected that these changes would be most distinct for slow loading rates, at levels below the loading rate used in the current study [59]. In the absence of a cytoskeletal disruptor, only a slight indentation was observed due to the applied force (Figure 5A), with no major indication of cytoskeletal remodeling under loading. In contrast, in the presence of acrylamide (Figure 5B) or cytoB (Figure 5C), the bead/tip significantly deformed the cell at an applied load of 3 nN. Since we observed a dramatic change in the actin filaments under these treatment conditions, cross-sectional images of single cells at 0, 1, 2 and 3 nN forces were acquired (Figure 6). For the untreated chondrocytes, the actin remained diffuse and spread slightly with increased indentation (Figure 6A). However, when the chondrocytes were treated with acrylamide (Figure 6B) or cytoB (Figure 6C), the actin initially appeared punctate, as noted above. With increased load, these punctate fluorescent spots spread out as the cell flattened, which potentially indicates that there is decreased connectivity/cohesion between the clusters of mCherry-actin. In contrast to the mCherry-actin, no major differences in the GFP-vimentin structures were observed between the treatments when under deformation. These findings further support the notion that mild disruption of the IFs may indirectly result in the disruption of the actin structure.

## Conclusions

The goal of this study was to systematically examine the effect of aging and cytoskeletal elements on mechanical properties of chondrocytes as a function of indentation (indentation-dependent mechanical properties). In addition, the microstructure of the different cytoskeletal elements in the presence and absence of cytoskeletal disruptors was examined under applied deformation. Our findings indicate that the elastic modulus of chondrocytes isolated from different aged bovines decreases with increasing deformation. In addition, similar trends were observed for chondrocytes isolated from cows of different ages (1 day, 5 yr and 12 yr old). We observed some significant differences with increased elastic properties and decreased intrinsic viscosity with healthy aging. We also systematically measured the contributions of actin and IFs on the indentation dependent mechanical properties of chondrocytes isolated from 1 day old bovine joints using cytoB and acrylamide, respectively. Our findings indicate that treatment with acrylamide resulted in a significant and almost complete loss of the indentation dependent response. In contrast, treatment of cells with cytoB diminished the elastic modulus measured at larger indentations, but not at small indentations. These findings suggest that IF integrity is a key contributor to the non-linear elastic modulus of chondrocytes. Furthermore, to better understand the effect of cytoskeletal disruptors, we imaged GFP-vimentin and mCherry-actin transfected chondrocytes in the absence or presence of an applied load and found that both treatments had a profound effect on disrupting the actin filaments, whereas acrylamide (specific disruptor of IFs) had no observable effect on the microstructure of the vimentin filaments. These results suggest that slight disruption of the IFs might indirectly alter the structure of the actin filaments, a novel phenomenon identified in chondrocytes. The combined results of this study underscore the importance of the cytoskeletal elements in the overall mechanical response of cells and improve our understanding of the overall mechanical properties of articular cartilage at the single cell level.

## Materials and Methods

### Isolation of Chondrocytes and AFM/microscopy Preparation

Bovine knee joints from 1 day, 5 year (yr) and 12 yr old cows were obtained from a local abattoir within 24 hours of slaughter (Figure 1A). Articular chondrocytes were isolated from the femoral condyles and cultured in Dulbecco's modified Eagle's medium (DMEM) with 10% fetal bovine serum (FBS) as previously described [60]. The cells were plated at high density and were examined by AFM or confocal fluorescent microscopy analysis within 36 hours. Measurements were acquired without passaging the cells since it has been previously shown that there is a significant increase in the elastic and viscoelastic moduli when the cells dedifferentiate in culture [61].

### Mechanical Stiffness of Chondrocytes Measured by AFM

Chondrocytes were plated on a number 1 circular glass slide (3 cm in diameter), incubated at 37°C for one hour and transferred to the AFM bioheater chamber (Aslyum Research, Santa Barbara, CA) in 3 ml of CO_2_ independent media. An Asylum MFP-3D AFM was coupled to a Zeiss Meta510 confocal microscope (Thornwood, NY). AFM microlevers (Model MSCT-AUHW, Veeco) with nominal spring constants between 0.01 and 0.05 N/m with a 10 µm polystyrene bead glued to the end of the cantilever (Figure 1B) were used to probe the chondrocytes. The spring constant of each cantilever was determined prior to each experiment based on the power spectral density of the thermal noise fluctuation [34]. Once the laser was aligned with the tip to produce a 0 V deflection, measurements were performed by lining up the probe with the center of the cell using visual inspection with the aid of the Zeiss microscope (Figure 1B). Contact with top of cell was determined in post-processing by identifying the point of inflection in the force-deflection curve as described below. Deformation was applied at 10 µm/s up to an indentation of ∼2000 nm, which corresponds to a deflection trigger point of ∼120 nm an approximately 20% cellular deformation (Figure 1C–D). To minimize potential artifacts associated with loading history and associated cytoskeletal remodeling contributing to measured responses, 30 consecutive force curves were collected with a 5 s rest between consecutive indentations, and the average load-deflection response was computed.

### Pointwise Modulus Analysis

The stress-strain curves were analyzed using a non-Hertzian technique as previously described [35], [36], [62], where no *a priori* assumptions about the linearity of the cell material properties were made. Briefly, the indentation data was analyzed to determine the point of contact between the AFM probe and the cell, using a non-linear least squares minimization algorithm (Levenberg-Marquardt solver) by fitting the indentation data with a bi-modal polynomial, where the region of pre-contact was described by a linear behavior and the region of post contact by a quadratic behavior [62]. Next, the position vs. deflection response in the post-contact region was used to compute the indentation force (F) vs. depth (D). The apparent ‘indentation dependent’ elastic modulus (E_<$>\scale 70%\raster="rg1"<$>_) was computed at each data point, <$>\scale 70%\raster="rg1"<$>:



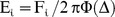



In classical linear elasticity theory (Hertz), *E_i_* is equivalent to *E*/2(1– v^2^). Rather than assume that *E_i_* is constant *a priori*, we solved the equation at each indentation data point, *i*, to obtain a pointwise apparent modulus. The term Φ (D) is an expression determined by the geometry of the AFM probe tip, which for a spherical indenter is:
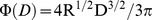
where R is spherical tip radius. The curve fits were performed using a non-linear least squares minimization (Nelder-Mead solver) with a custom Matlab program. The mean indentation-dependent modulus was computed for each group using linear interpolation. The values reported are the average and standard deviations of n = 10–15 cells per condition.

### Viscoelastic Properties of Chondrocytes Measured by AFM

Cells were prepared and aligned under the cantilever as described above (Figure 1B). A 1500 nm indentation was applied at a tip velocity of 10 µm/s resulting in an ∼15% applied deformation. Deformation was held constant for 10 s and the resulting relaxation in load was measured prior to disengaging the AFM probe (Figure 1E). This same procedure was repeated 10 consecutive times with a 5 s interval between successive curves. The resulting force vs. time relaxation response was fit to a standard linear solid viscoelastic model [34], yielding an relaxation modulus (E_r_), relaxation time constant under constant load (τ_σ_) and relaxation time constant under constant deformation (τ_ε_), with a Poisson’s ratio (v) taken to be 0.35 [61], [63]–[65] for each curve. The values reported represent an average of 10 consecutive loading curves. These properties were used to compute the equilibrium modulus (E_Y_), instantaneous modulus (E_o_) and viscosity (μ) of the cell. Viscoelastic curve fits were performed using a non-linear least squares minimization (Nelder-Mead solver) with a custom Matlab program. These measurements were performed on chondrocytes isolated from 1 day old bovine cartilage. Viscoelastic properties were measured on untreated cells as well as cells subjected to cytoskeletal disruption as described below. The values reported are the average and standard deviations of 10 cells per condition.

### Disruption of the Cytoskeletal Elements

Cytochalasin B and acrylamide were used to disrupt actin and IFs, respectively. Immediately after isolation, chondrocytes were cultured in media containing 5 mM acrylamide for 24 hrs, as previously described [48]. On the day of testing, cells were transferred to the bioheater chamber and were incubated with CO_2_ independent media also containing 5 mM acrylamide. CytoB treatment for actin disruption was performed on un-treated cells on the day of AFM testing. Cells were transferred to the bioheater chamber and incubated in CO_2_ independent media and 5 µM cytoB for 1 hr prior to AFM measurements [66].

### Transfection of Chondrocytes with mCherry-actin and GFP-vimentin

The GFP-vimentin expression cassette described previously [22], and the mCherry-Actin expression cassette (Clontech, Mountain View CA), were independently subcloned into the pCCLc lentiviral vector. Infectious, but replication-defective lentiviral particles were packaged in 293T cells by co-transfection with pMDG-VSVG envelope and pCMV-d8.9 packaging vectors, at the Vector Core Facility of the UC Davis Medical Center. The lentiviral titer was determined by qPCR (Applied Biological Materials, Richmond BC Canada). Chondrocytes were transduced with the lentivirus as described previously [67]. Briefly, cells seeded at 1×10^5^ cells/cm^2^ in DMEM/10%FBS were allowed to adhere; after 4 hrs, 1 µg/ml polybrene was added, followed by lentiviral particles at an MOI of 5 for both the GFP-vimentin and mCherry-Actin constructs. Cells were incubated with virus overnight, then thoroughly washed with DMEM/10%FBS media, and subsequently used for the confocal AFM studies.

### Imaging Labeled Chondrocytes with CFM when Under Compression by AFM

GFP-vimentin and mCherry-actin labeled cells were treated with Cytochalasin B and acrylamide as described above and were tested on a combined Asylum Research MFP3D AFM and Olympus America FV1000 confocal fluorescent microscope. Images were acquired at excitation wavelengths of 488 nm (Argon laser; GFP-vimentin) and 543 nm (HeNe laser; mCherry-actin) and emission wavelengths of 505 nm (GFP-vimentin) and 575 nm (mCherry-actin). Cells were imaged in the absence of compression by the AFM tip and then in increments of 1.5 nN of applied force. All acquired images were analyzed using the NIH Image J program. No significant remodeling of the actin and vimentin filaments was observed under repetitive loading.

### Statistical Analysis

Statistical analyses of the elastic properties were performed with repeat measure ANOVA, with age or treatment as independent variables and indentation as a repeat measure factor. The viscoelastic properties were analyzed with a general linear model nested design ANOVA with age (neonatal, adult, geriatric) and treatment (control, acrylamide, cytoclasin) taken as nested variables. LSD Post Hoc tests were performed for each variable, with p<0.05 considered significant. Analysis was performed in STATISTICA (StatSoft Inc, Tulsa, OK).
